# Childhood and adolescent cancer in Germany – an overview

**DOI:** 10.25646/11438

**Published:** 2023-06-14

**Authors:** Claudia Spix, Friederike Erdmann, Desiree Grabow, Cécile Ronckers

**Affiliations:** German Childhood Cancer Registry Division of Childhood Cancer Epidemiology Institute of Medical Biostatistics, Epidemiology and Informatics University Medical Center of the Johannes Gutenberg University Mainz

**Keywords:** CANCER, CHILDREN, ADOLESCENTS, GERMANY, EPIDEMIOLOGY, PROGNOSIS, LONG-TERM FOLLOW-UP

## Abstract

**Background:**

Childhood and adolescent cancer constitutes only a very small fraction of the cancer cases in Germany and throughout the world, but it is the most frequent cause of disease-related death in children. The diagnostic spectrum differs markedly from that of adults. More than 90% of all cases of childhood and adolescent cancer in Germany are treated according to centralised protocols or in therapy studies.

**Methods:**

The main epidemiological data for this group are collected by the German Childhood Cancer Registry (GCCR) since 1980. Based on this data, three typical diagnoses and their incidence and prognosis are described in exemplary manner: Lymphoid leukaemia (LL), astrocytoma and neuroblastoma.

**Results:**

Approximately 2,250 new cancers are diagnosed in children and adolescents under the age of 18 in Germany every year. In this age group, leukaemia and lymphoma account for almost 50% of all new cancer cases, predominately acute forms. Overall, the prognosis is considerably better than in adults.

**Conclusions:**

There is relatively little consistent evidence available on external factors as risk factors for childhood cancer, despite decades of research. For LL, the immune system and infections are assumed to play a role, as early training of the immune system appears to be protective. To an increasing degree, research is identifying genetic risk factors for many types of childhood and adolescent cancer. The therapy is sometimes very intensive and leads to a variety of late effects for at least 75% of the survivors, which may occur soon after the primary diagnosis, but also decades later.

## 1. Introduction

Childhood and adolescent cancer constitutes only a very small proportion of cancer cases in Germany and throughout the world: The approximately 2,250 newly diagnosed cases in children and adolescents in Germany each year account for less than half a percent of the total number of cancer cases [1, 2]. From a different point of view, however, cancer is the most frequent cause of disease-related death in children aged 1 year and older. Since pediatric cancer survivors’ life expectancy is long and late-effects can last for or manifest during their entire lifetime, the relevance of the topic is larger than it initially seems based on the number of patients.

One in 370 girls and one in 310 boys are diagnosed with cancer before their 18th birthday, about half of them before school age. The spectrum of diagnoses and the proportional distribution of the diagnoses differ considerably from what is seen in adults: Some diagnoses which are relatively common in childhood and adolescence are not or rather rarely seen in adulthood (the most frequent diagnosis at an adult age which also occurs in children, i.e. lymphoma, accounts for about 4% of adult cancer cases [2]); conversely, none of the diagnoses that are particularly frequently observed in adults (carcinomas of breast, prostate, bowel and lung) play a major role in children and adolescents [1]. The risk factors discussed for a large proportion of cases in adulthood generally do not apply.

The German Childhood Cancer Registry (GCCR) began to record the essential epidemiological data in 1980; here we briefly introduce the registry. Based on registry-data, three typical forms of childhood cancer along with their incidence and prognosis are herein presented in exemplary manner: Lymphoid leukaemia, astrocytoma and neuroblastoma. Furthermore, we will present a brief outline of what is known on causes and risk factors.

The therapy protocols (guidelines for diagnosis and treatment) in paediatric and adolescent oncology are centrally organised per diagnostic group throughout Germany, and often even across countries within Europe, and they are monitored closely. This has a tradition in German paediatric oncology, and is also necessary due to the small number of cases. This structure aims to achieve continuous improvement of the treatment and thus of the survival probability [3]. As meanwhile the prognosis has become very good for a considerable proportion of affected children and adolescents, many survivors have a life-long risk of late effects which can be attributed to the disease and/or the intensive therapy. The therapeutic improvements lead to an increase in the number of survivors with health care needs, ensuing specific and new challenges for the health care system. The most important aspects of research on late effects and long-term follow-up care are presented herein.

## 2. Data basis

The German Childhood Cancer Registry (GCCR) was founded in 1980 at the University Medical Centre of the Johannes Gutenberg University Mainz, with support of the predecessor societies of the Society for Paediatric Oncology and Haematology (GPOH). At the time the GCCR was founded, nationwide cancer registration was not yet established in Germany. The GCCR is one of the largest and oldest childhood cancer registries in the world. The GCCR contributed the largest single data set to the current worldwide overview ‘International Incidence of Childhood Cancer, Volume 3 (IICC3)’ of the IARC (International Agency for Research on Cancer of the WHO) [4]. The reports in Germany are almost exclusively provided by centres for paediatric and adolescent oncology. The guideline on paediatric oncology (KiOn-RL) of the Federal Joint Committee (G-BA) recommends that paediatric and adolescent oncology centres inform the affected families of the option to be reported to the GCCR [5]. Currently, there are still approximately 60 specialised centres in Germany, although there is an ongoing trend towards further centralisation [1, 6]. With the exception of a few types of cancer in older adolescents, which are not predominantly treated in paediatric and adolescent oncology centres, registration was mostly complete since about 1987, which is also reflected in internationally comparable rates of new cases (incidence rates).

Currently most paediatric and adolescent oncology centres treating these patients report a newly diagnosed child to the GCCR on a paper form. This system is being transitioned to a digital reporting procedure compatible with that of the clinical-epidemiological state cancer registries. These electronic notifications are transferred by the digital tumour documentation systems of the centres, which also handle the mandatory reporting to the state cancer registries this task for all oncology departments of a clinic. A written informed consent from the custodians (parents) and/or patients is required so the German Childhood Cancer Registry can receive and process the data. By law, the state cancer registries are in charge of documenting all adult cancer patients from age of 18. The cooperation between the state cancer registries acting on a legal basis and the GCCR is a challenge which is currently being addressed. The joint goal is avoiding gaps in registration and enabling long-term follow-up across all ages.

The GCCR provides important functions for the German health system. In addition to regular annual reports [1] these include contributions to ‘Cancer in Germany’ of the RKI [2], contributions to governmental health reporting (e.g. [7]) and the implementation of research projects for various institutions (e.g. Federal Office for Radiation Protection). Investigations of suspected local clusters are conducted on request. The German Childhood Cancer Registry represents Germany in the international reporting of childhood and adolescent oncology, such as the IICC or EUROCARE [8].

Advances in therapy have led to long-term survival for more than 80% of all childhood and adolescent cancer patients. As a consequence, long-term follow-up has become as important in the tasks of the GCCR as the registration of new patients. With regard to long-term follow-up, the focus of the registry is on vital status and the recording of subsequent cancer cases, which occur considerably more frequently after a first cancer diagnosis in childhood and adolescence compared to cancer in the general population at the same age [9].

In addition to basic registration (recording new cases, recording subsequent cases, and deaths), the GCCR contributes to a variety of research projects – on its own, in cooperation, and by contributing logistics and/or data. More information is available in the annual reports and at www.kinderkrebsregister.de [1].

The data presented in the following are based on publicly accessible sources. Specifically these include the annual report of the German Childhood Cancer Registry (GCCR) [1], the regular publication ‘Cancer in Germany’ of the Robert Koch Institute (RKI) [2], the publications of the EUROCARE group [8], as well ‘International Incidence of Childhood Cancer (IICC)’ [10].

In addition, there are a number of reviews and synoptic publications, mainly from the Society for Paediatric Oncology and Haematology (GPOH) or in collaboration with researchers and physicians of the GPOH, such as [3].

## 3. Childhood and adolescent cancer

### 3.1 Overview

The German Childhood Cancer Registry began registration in 1980, and registration has been complete for most diagnoses since about 1987 judging by international standards. In 1991, registration was extended to include former East Germany. From 2009, registration was extended from age 0–14 to 0–17. A total of 74,258 new cancer cases were registered from 1980 to 2021. Given the current total population, this is equivalent to approximately 2,250 newly diagnosed cases (incident cases) in children and adolescents under 18 years of age in Germany every year [1].

Incident cases in children are grouped and reported according to the International Classification of Childhood Cancer (ICCC), a fundamentally different classification than that used for adults; the ICCC3 version based on the ICD-O-3 is currently in use [11, 12].

**Figure 1:**
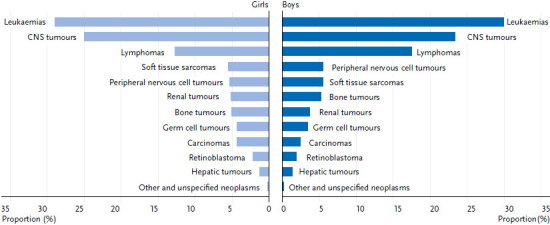
Percentages of the most frequent cancer diagnoses in children and adolescents under 18 years of age in Germany by sex (2010–2019) Source: German Childhood Cancer Registry, [1]

Systemic malignant neoplasms, i.e. leukaemia and lymphoma and related diseases, account for almost 50% of all new cases in children and adolescents under the age of 18 (Figure 1). Acute forms predominate. Chronic forms, such as chronic lymphoid leukaemia, which is the most common form of leukaemia in adulthood, are diagnosed very rarely in children (approx. 10 cases per year in Germany).

With regard to solid tumours, sarcomas, blastomas, and some other forms of neoplasms predominate. Carcinomas, which predominate in adulthood, occur very rarely and when they do, mainly in older adolescents. Subsequent cancer represents a known late effect and some of the further cancers, so-called ‘subsequent primary neoplasms (SPN)’ may already occur in childhood and adolescence, for example thyroid carcinomas and skin tumours [9]. A SPN is a new subsequent cancer that is different from the initial diagnosis and also is not a relapse of the initial diagnosis. It can be an independent event, but can also be a consequence of an underlying genetic variant and/or a consequence of the therapy of the initial disease.

### 3.2 Incidence rates, age distribution and survival probability of three exemplary cancer diagnoses

Despite the current survival probabilities being generally high (see below), cancer in childhood and adolescence remains the most frequent disease-related cause of death in this age group.

In this paper we present three selected diagnoses: lymphoid leukaemia is the single most frequent diagnosis, astrocytoma is the most common tumour of the central nervous system (CNS) and neuroblastoma is a typical malignant disease in early childhood (aside from CNS tumours this is the most frequent solid tumour in children).

#### Lymphoid leukaemia

**Figure 2:**
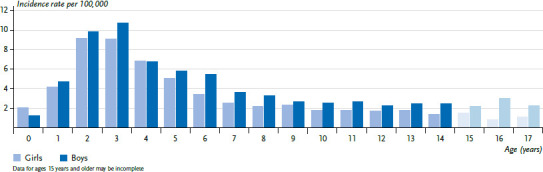
Leukaemia incidence rates in children and adolescents in Germany by sex and age (2010–2019) Source: German Childhood Cancer Registry, [1]

Lymphoid leukaemias (LL, ICCC3 group I(a)) account for 75% of all leukaemias, myeloid and myeloproliferative diseases (ICCC3 class I) in childhood and adolescence. Other important diagnoses in this class include acute myeloid leukaemia (I(b)) and the myelodysplastic syndrome (I(d)). A total of 98% of all LL are B-precursor cell leukaemias (I(a)1) and thus acute lymphoid leukaemias. One in 1,700 girls (approx. 210 new diagnoses/year) and one in 1,350 boys (approx. 280 new diagnoses/year) are diagnosed before their 18th birthday. The average age at diagnosis is between the 5th and 6th birthday, with children between their 1st and 6th birthday being affected particularly frequently. However, the disease also manifests in infants (‘infant leukaemia’) and older adolescents. Boys are affected more often than girls in almost all age groups except for the first year of life (Figure 2) [1].

The incidence rate in Germany, and also in Europe, has increased slightly but steadily since the 1980s, with a plateau being reached since about 2005 [1, 13]. The incidence rate in the former GDR was about 20% lower than in the West of Germany, but the incidence rates have converged since about 1997. The different living conditions in the former GDR, such as the higher vaccination rate, higher number of births and thus larger number of siblings, more crowded living conditions and more frequent and earlier care in day-care centres as compared to the ‘West’ are presumed to have had an overall protective effect. This would be consistent with studies, including some from other countries, but it cannot be definitively proven [14, 15].

**Figure 3:**
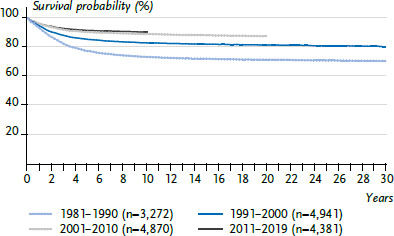
Survival probability after lymphoid leukaemia in children and adolescents under 18 years of age in Germany by decade of diagnosis Source: German Childhood Cancer Registry

The 15-year survival probability is now 90% (Figure 3). The 5-year survival of 92% in Germany is slightly above the European average of 90% [1, 8]. Survival has improved significantly since the 1980s, when the 15-year survival probability was 64%, to today’s 90% 15-year survival probability, with most of the progress having been achieved before 2000. The incidence of SPN after LL is average compared to the incidence after all childhood cancers; LL in turn are not particularly frequent as SPN. In contrast, acute myeloid leukaemias (ICCC3 I(b)) are rather common SPNs, and in the first 5 years after an initial cancer diagnosis they are the single most frequent subsequent cancer diagnosis [1, 9].

#### Astrocytoma

**Figure 4:**
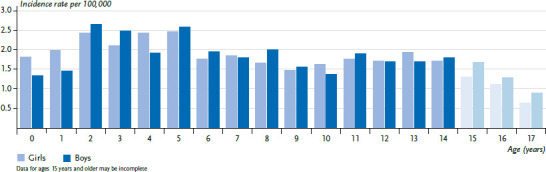
Astrocytoma incidence rates in children and adolescents in Germany by sex and age (2010–2019) Source: German Childhood Cancer Registry, [1]

Astrocytoma (ICCC3 III(b)) accounts for 45% of all reported CNS tumours (ICCC3 III). About half of the reported astrocytoma diagnoses were reported as malignant or as ‘uncertain’ respectively. Of all reported CNS tumours, 56% are malignant and astrocytomas account for 38% of these. One in 3,180 girls (approximately 110 new diagnoses/year) and one in 3,150 boys (approximately 120 new diagnoses/year) are diagnosed with the disease before their 18th birthday. Girls and boys are affected more or less equally, and there is no marked age dependence (Figure 4). In Germany, an increase in incidence was observed until about 2005: We assume that the non-malignant brain tumours were not completely reported earlier and that the increase in incidence reflects the efforts to improve completeness. By now, the incidence rates in Germany are comparable to those in the Nordic countries, whose registries are considered to have practically complete coverage [4].

**Figure 5:**
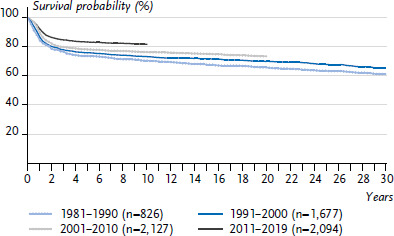
Survival probability after astrocytoma in children and adolescents under 18 years of age in Germany by decade of diagnosis Source: German Childhood Cancer Registry

The current long-term prognosis of 81% for astrocytomas is rather good compared to other CNS tumours (Figure 5). The 5-year survival in Germany is 84%, which is slightly above the European average of 80% [16]. In the most recent 1–2 decades, further improvements in survival probability have been achieved. The risk of SPN after astrocytoma is below average compared to the incidence after all childhood cancers. CNS tumours account for almost a quarter of SPNs observed in the first 30 years after the primary diagnosis, more than half of those are meningiomas (ICCC3 III(e)5), astrocytomas are the second most frequent ones [1, 9].

#### Neuroblastoma

**Figure 6:**
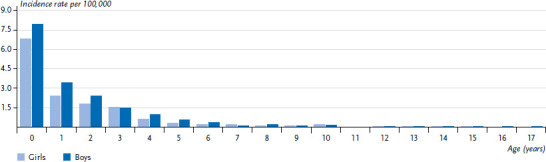
Neuroblastoma incidence rates in children and adolescents in Germany by sex and age (2010–2019) Source: German Childhood Cancer Registry, [1]

Neuroblastoma (ICCC3 IV(a)) is part of the group of embryonal tumours, which are mostly diagnosed very early in life, in rare cases even prenatally. Other embryonal tumours in childhood include nephroblastomas (ICCC3 VI(a)), hepatoblastomas (ICCC3 VII(a)), retinoblastomas (ICCC3 V(a)) and medulloblastomas (ICCC3 III(c)1) [17]. One in 6,850 girls (approximately 50 new diagnoses/year) and one in 5,300 boys (approximately 70 new diagnoses/year) are diagnosed with neuroblastoma by their 18th birthday [1]. Neuroblastoma occurs almost exclusively before school age, most frequently in the first year of life, and most frequently in the first months of life. Overall, boys are affected 40% more often (Figure 6).

A large-scale study was conducted in Germany from 1995 to 2001 to investigate whether the prognosis can be improved through screening, by diagnosing neuroblastoma before it becomes metastatic. It turned out that the number of children with metastases could, unfortunately, not be reduced, while a relatively large number of tumours, which otherwise would have regressed spontaneously without diagnosis and treatment, were additionally diagnosed by screening. Accordingly, some children were thus possibly overtreated [18, 19]. The German study focused on children after their first birthday, a similar project with younger children in Canada produced the same results. The contribution of the German Childhood Cancer Registry was crucial for this project [20, 21].

**Figure 7:**
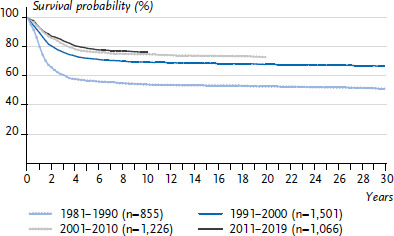
Survival probability after neuroblastoma in children and adolescents under 18 years of age in Germany by decade of diagnosis Source: German Childhood Cancer Registry

There was considerable progress made in therapy in the 1980s and early 1990s, but no major further improvements have been achieved since. The 15-year survival is now at 77% (Figure 7). The 5-year survival of 80% in Germany is clearly above the European average of 75% [1, 8]. The prognosis depends very much on age and cancer stage at diagnosis: While almost all children under 18 months of age with a localised tumour survive, and chemotherapy and radiotherapy can sometimes be omitted from the treatment, the prognosis for children 18 months of age and older, relatively many of whom are already diagnosed at a metastatic stage, is unfortunately still slightly below 50%, despite decades of research and improvements in therapy [22]. The risk of SPN is below average compared to the incidence after all childhood cancer diseases; neuroblastomas almost never manifest as SPN [1].

### 3.3 Causes and risk factors

Despite many years of major international research initiatives, there are relatively few consistent insights into the causes and risk factors of childhood cancer as far as it concerns external factors; in particular, study results on environmental factors are very inconsistent. As far as currently known, external risk factors, i.e. potentially alterable risk factors, are responsible for at most a small fraction of the cases in children.

In leukaemias and CNS tumours, radiation exposure and exposure to pesticides play a role in a small number of cases [23, 24]. For leukaemias, an increased risk by birth weight (20% increase in risk per kg of extra weight or increased risk for children above 4000g) has been reported in several studies [25]. From many different perspectives, a role of the immune system and infections has been reported, at least for LL, especially for the most common subtype B-precursor cell leukaemia. Early training of the immune system (in the first years of life) lowers the risk somewhat. Immune system training is associated with breastfeeding, vaccination, many social contacts with humans and animals (which in turn is associated with social status (higher or lower social status depending on the social background), large families or living with older siblings, institutional child care and cramped living conditions) and infections experienced in early life [26–29]. In some cases, an untrained (‘naïve’) immune system seems to respond with leukaemia to an infection, a phenomenon demonstrated for influenza epidemics [30–32].

Little is known about risk factors for astrocytomas. With regard to astrocytomas and meningiomas, cases occur more often after prior therapeutic cranial irradiation [9, 33].

Many embryonal tumours, especially nephroblastomas, have been reported to be associated with congenital malformations [34]. Hardly anything is known about risk factors specifically for neuroblastoma. The emergence of neuroblastoma cells is an ongoing research issue [35, 36].

Strong associations of cancer risk in childhood and adolescence have been identified with a growing number of rare genetic syndromes, some of which are very specific for certain combinations of rare syndromes and cancer types [37–44]. For example, patients with RASopathies (a specific group of rare genetic syndromes such as Noonan syndrome), have ten times the cancer risk than expected, an approximately 30-fold risk was observed for Beckwith-Wiedemann syndrome and about 40-fold for Fanconi anaemia. In recent years, research focussed on the search for other previously unknown genetic risk factors, which seem to play a greater role in childhood cancer than previously assumed [37, 44]. These genetic syndromes also seem to be partly responsible for patients experiencing multiple subsequent cancers in their lifetime. According to the most recent findings, the increased risks of SPN after childhood and adolescent cancer due to chemotherapy or radiotherapy of the initial disease may also be modified by predisposing genetic factors [45].

### 3.4 Therapy

More than 90% of all patients with childhood and adolescent cancer in Germany are treated according to the nationally standardised protocols or in therapy studies centrally organised by the GPOH. Mostly initiated in the 1970ies, this system involves the GPOH appointing experts to diagnostic groups, who then develop and further improve the therapy protocols and monitor compliance with the protocols in the paediatric oncology centres [3, 46]. Due to the rarity of each single diagnosis, it is of considerable importance for the centres to have knowledgeable consulting specialists for each patient during the treatment within the framework of this system. A wide range of therapy elements are used for childhood and adolescent cancer, including surgery, cytostatic chemotherapy, radiotherapy, stem cell therapy and antibody therapies [46–48]. The therapy regimens have become increasingly differentiated over the past decades, so that today the therapy is largely tailored to the individual patient. For this reason, further therapy research now requires international cooperation for almost all diagnostic groups. The GPOH cooperates with the working groups of the SIOP (International Society of Pediatric Oncology) and the SIOPE (SIOP Europe). Especially in the case of diagnoses where further improvements in prognosis are difficult to achieve, the focus of clinical research is increasingly shifting towards reducing the short- and long-term physical consequences of the therapy on healthy tissue (toxicity), while still maintaining the same level of survival.

### 3.5 Long-term survival and late effects

Since the prognosis is good and the life expectancy of the affected children and adolescents is long, the long-term follow-up after cancer in childhood and adolescence plays a major role [49]. The sometimes very intensive therapy leads to considerable late effects on the body, including increased mortality, sometimes soon after the primary diagnosis, sometimes decades later [50–52]. The international literature assumes that at least 75% of all survivors are affected by late effects [53]. Some late effects are known from adult oncology, but in many cases the fact that a body was exposed to chemotherapy or radiotherapy during growth plays a special role. Late effects can affect all aspects of health beyond the notable SPN risk, as exemplified by relatively early onset of heart failure (cardiomyopathy), hearing impairment, fertility and other endocrine (hormonal) disorders. Irradiation of the brain can also lead to cognitive impairment [54–56].

The world wide data on late effects for former patients up to a current age of about 50 years is relatively good, but unfortunately relatively few data are available for patients beyond this age [57, 58]. This is due to the fact that before about 1970 the prognosis of childhood cancer was still very poor, but also because hardly any systematically collected data and major long-term cohorts are available from before this: the German Childhood Cancer Registry was founded in 1980; with some exceptions, the earliest registries, cohorts and data collections in Europe and the USA did not start much before the 1970s [59–61].

In Germany, as in many other countries, the transition of long-term survivors from paediatric and adolescent oncology to community-based care as they reach adulthood is a major problem for the survivors. Ongoing projects on healthcare address many different aspects, such as the Verskik project [62], which examines the extent of survivors’ current usage of the healthcare system and whether the care meets the standards recommended in the guidelines. A wide range of GPOH initiatives aim to provide survivors with care better tailored to their needs and to improve the pertinent guidelines [63]. At the European level, there are established structures in some countries [64, 65] and initiatives to improve it under the umbrella of PanCare [66].

## 4. Conclusion

Childhood and adolescent cancer is rare and rather special in terms of diagnoses and research topics. Still there are some overlaps with the registration of and research efforts on the much more frequent cancer cases in adulthood. Germany has special established structures for the care and registration of childhood and adolescent cancer: the Society for Paediatric Oncology and Haematology (GPOH) and the German Childhood Cancer Registry (GCCR).

The close cooperation with the specialised centres for paediatric and adolescent oncology ensures complete reporting up to the age of 15, but not necessarily beyond this age, which leads to less than complete reporting of cases in older adolescents from the age of 15 and certain diagnoses, as some of these older patients are being treated in general oncology centres. The reporting channels to the GCCR have not yet generally been extended to this group. The implementation of electronic reporting out of the tumour documentation systems of the hospitals, analogous to the procedure adopted by the state cancer registries, is expected to solve this problem.

The 10-year survival probability of 85% is much better than the cancer survival for older age groups (less than 50%) [1, 2]. The 10-year prognosis improved from overall 66% and 74% in the 1980s and 1990s, respectively, to 85% in the most recent decade [1]. For many types of childhood and adolescent cancer, a plateau has by now been reached, where further improvement seems unlikely for now. In contrast to adults, individual lifestyle-related risk factors play only a minor role. A steadily increasing number of genetic risk factors for childhood and adolescent cancer have been identified through research in recent years, which opens up new paths for diagnostics and therapy, perhaps even for screening.

The focus on long-term follow-up of the many long-term survivors and their broad range of symptoms and late effects is a challenge for paediatric and adolescent oncology to get involved in healthcare research beyond the traditional inpatient clinical care to improving long-term care.

## Key statement

- Approximately 2,250 children under the age of 18 are newly diagnosed with cancer every year in Germany.
- In 1980, the German Childhood Cancer Registry (GCCR) began to collect and report epidemiological data on childhood and adolescent cancer.
- The diagnostic range in childhood and adolescence differs considerably from that in adults.
- To an increasing degree, genetic risk factors are being identified for many types of childhood and adolescent cancer.
- The prognosis is considerably better than for adult cancer, making late effects an important issue.
